# Dentinogenesis Imperfecta and Caries in Osteogenesis Imperfecta among Vietnamese Children

**DOI:** 10.3390/dj9050049

**Published:** 2021-04-27

**Authors:** Huong Thi Thu Nguyen, Dung Chi Vu, Duc Minh Nguyen, Quang Dinh Dang, Van Khanh Tran, Hung Le, Son Minh Tong

**Affiliations:** 1 School of Odonto-Stomatology, Hanoi Medical University, Hanoi 100000, Vietnam; thuhuongnguyen@hmu.edu.vn; 2 Department of Endocrinology, Metabolism and Genetics, Vietnam National Children’s Hospital, Hanoi 100000, Vietnam; dungvu@nch.org.vn; 3 Division of Research and Treatment for Oral Maxillofacial Congenital Anomalies, Aichi Gakuin University, Aichi 464-8651, Japan; 4 Faculty of Dentistry, Hanoi University of Business and Technology, Hanoi 100000, Vietnam; Ddquang91@gmail.com; 5 Center for Gene and Protein Research, Hanoi Medical University, Hanoi 100000, Vietnam; tranvankhanh@hmu.edu.vn; 6 Dong Da General Hospital, Hanoi 100000, Vietnam; Lehungphd68@gmail.com

**Keywords:** caries, dentinogenesis imperfecta, osteogenesis imperfecta

## Abstract

Osteogenesis imperfecta (OI) is a genetic disorder characterized by increased bone fragility and low bone mass, caused mainly by mutations in collagen type I encoding genes. The current study aimed to evaluate dentinogenesis imperfecta (DI), oral manifestations and caries status of OI children. Sixty-eight children (41 males, 27 females) aged from 3 to 17 years old (mean 9 ± 4.13) participated in the study. Participants were classified into three OI type groups (I—2 cases, III—31 cases and IV—35 cases). Clinical examination and an orthopantomogram were used to obtain prevalences and associations of DI, caries status, malocclusion, crossbite, open bite, eruption, impaction and missing teeth with OI. The prevalence of DI among OI patients was 47.1%, more common in OI type III than type IV. The yellow-brown discoloration type was more vulnerable to attrition than the opalescent-grey one in the primary dentition. OI seemed not to have a high risk of caries; the prevalence of caries was 69.1%. A high incidence of malocclusion, crossbite and open bite was observed. In-depth oral information would provide valuable data for better dental management in OI patients. Parents and general doctors should pay more attention to dental care to prevent caries and premature tooth loss.

## 1. Introduction

Osteogenesis imperfecta (OI) is a genetic disorder characterized by increased bone fragility, low bone mass, and growth deficiency mostly caused by mutations in collagen type I encoding genes (COL1A1 and COL1A2) [1]. Clinical severity is a continuum, varying widely from prenatal lethality, extreme bone fragility to nearly asymptomatic without bone fracture [2]. Based on disease severity, the most commonly used classification by Sillence et al. [3] divided OI into four clinical types. Type I shows a non-deforming OI with a deficiency in the quantitation of normal collagen molecules. In contrast, type II to IV show a decreasing severity of deformity and growth retardation from lethal (type II) and severe (type III) to moderate (type IV) as the result of altering collagen structure. OI’s other characteristics include blue sclera, joint laxity, hearing loss, and oral manifestations [1].

Approximately 50% of OI children present with dental abnormalities in various degrees [4], and dentinogenesis imperfecta (DI) is the most prominent oral characteristic of OI patients [5,6]. According to the Shields classification [7], there are three types of DI; only DI Shields type I is associated with OI, whereas DI type II and III are non-syndromic. Clinical signs appear as opalescent blue-grey or yellow-brown discoloration of the teeth. Due to the defection of the underlying dentin (type I collagen), teeth present radiographically with poor mineralization density, bulbous crowns, cervical constriction, short roots, and pulp canal obliteration. Previous studies showed a varying prevalence of DI among OI patients, depending on population, such as 19% in Norwegians [8], 20.4% in Egyptians [9], 24.7% in Danish [10]; or types of OI, from 31% in type I OI to 86% in OI type III [11]. It should be noted that even though DI could be clinically evident in both dentitions, this disorder is more frequent and severe in the primary teeth [12]. Thuesen et al. [10], in a Danish adult study, reported that only 1.8% of OI type I patients presented with DI. However, O’Connell and Marini [13] revealed that more than 80% of OI English children presented with DI in the primary dentition.

Although DI type I is caused by mutations affecting collagen formation, not dentin proteins (dentin sialophosphoprotein) [14] like in DI type II and III, the dentin is similarly affected [13]. These include reduced numbers of tubules and irregular tubular morphology [15]. The enamel–dentin junction is also altered, significant attrition can spontaneously occur, and teeth tend to be prematurely lost even though the enamel layer is normal [16]. Therefore, OI patients seem to be a high-risk group to caries. A study conducted on a large sample of OI patients (319 participants) revealed that OI’s presence increases the probability of caries [17]. However, another one reported that caries’ prevalence was similar to the general population [18].

Other oral problems associated with OI are malocclusion, hypodontia and impacted teeth [8,19,20]. Numerous studies reported Angle Class III malocclusion, anterior and posterior crossbites, open bites, hypodontia, and impacted teeth in OI patients [21,22,23,24]. Jensen and Lund [25] revealed that more severe OI patients were associated with more severe craniofacial abnormalities. Furthermore, since bisphosphonate (BP) treatment remains the first line medical management, particular attention should be paid to the risk of developing osteonecrosis of the jaw (ONJ) and the management of this possible complication [26,27,28]. However, the literature confirmed the lack of BP-related ONJ occurrence in the pediatric population suffering OI and treated with BP [29].

For these reasons, in-depth oral information in both dentitions and different OI types would provide valuable data for better dental management in OI patients. Unlike bone, the dentine structure’s defects cannot be reversed or lessened [30]. Early and appropriate treatments could help OI patients maintain their teeth as long as possible and obtain a better life quality. The current study aimed to describe clinical and radiographic oral manifestations in Vietnamese osteogenesis imperfecta patients.

## 2. Materials and Methods

### 2.1. Participant Recruitment

This study was approved by the Ethics Board of Hanoi Medical University (no. NCS19/HMU-IRB). Written informed consent was obtained from parents of all under 18-year old patients. All patients diagnosed with OI in a Hospital of Pediatrics from 12/2018 to 6/2020 were recruited. OI diagnosis was mainly based on clinical and radiographic findings, including bone fractures, bowing deformities of long bones, growth deficiency, macrocephaly, flat midface and triangular facies, dentinogenesis imperfecta, hearing loss, chest wall deformities, barrel chest, scoliosis, kyphosis [31,32], osteopenia, long-bone bowing, under-tubulation and metaphyseal flaring, gracile ribs, narrow thoracic apex, and vertebral compressions [33]. Positive family history was used for diagnosis. Uncertain cases were confirmed by collagen type I gene analysis (COL1A1 and COL1A2). According to OI types, patients were classified into four groups based on the Sillence classification [3]. Since OI type II is a prenatal lethality, there were no patients recruited in that group. Patients who (1) had bone deformity without OI, or (2) were aged under three years old or had no teeth, (3) or received orthodontic treatments were excluded.

Sixty-eight children (41 males, 27 females) aged from 3 to 17 years old (mean 9 ± 4.13) participated in the study (Table 1).

### 2.2. Clinical and Radiographic Examinations

Oral examination was performed by experimented dentists. Recorded oral features consisted of tooth discoloration, attrition, caries, occlusion (open bite, crossbite, Angle malocclusion), delayed eruption, and missing teeth. Angle malocclusion was determined from the first permanent molar relationship. Children who lost first permanent molars or did not fully achieve the first permanent molar relationship were not assessed. Normal tooth eruption time was referenced by Holt, Roberts [34]. The delayed or early eruption was detected if there was more than a 12-month deviation from normal norms [34].

Due to the incorporation of very young patients, only 52 orthopantomograms were obtained; combinations of periapical radiographs were taken in the other 16 patients. Impacted teeth, hypodontia, ectopic teeth of upper molars, low mineralization density, bulbous crowns, cervical constriction, short roots, and pulp canal obliteration were carefully assessed.

#### 2.2.1. Caries Diagnosis

Caries was diagnosed based on the International Caries Detection and Assessment System (ICDAS) scores [35], with code 3, 4, 5, 6 as caries and code 0, 1, 2 for no caries [36]. Decayed, Missing and Filled Teeth (DMFT, dmft) indices were used for assessing caries status [37]. Since a high prevalence of missing teeth due to breaking was assumed and it is challenging to distinguish missing teeth caused by caries or breaking, the index missing teeth (mt, MT) was eliminated to avoid skewing the actual estimate of missing teeth due to caries. The new dft (DFT) scores were calculated by the sum of dt (DT) and ft (FT).

#### 2.2.2. Dentinogenesis Imperfecta Diagnosis

Dentinogenesis imperfecta was diagnosed based on clinical features and radiographic findings [5]. Clinical signs included soft, blue-to-brown translucent discolored teeth, significant spontaneous attrition, and premature tooth loss. Radiography could present with poor mineralization density, bulbous crowns, cervical constriction, short roots, and pulp canal obliteration [16].

### 2.3. Statistical Analysis 

All statistical analyses were performed by SPSS 20 (IBM, NY). The significance level was set at 0.5. Since only two patients were recruited, only descriptive analysis was given for the type I OI group. Calculating differences between OI type III and IV and dentitions in the prevalence of DI, caries, malocclusion was performed by chi-square test. Analysis of variance (ANOVA) was used to compare dft (DFT) indices between dentitions. Once the ANOVA significant difference was found, an LSD pairwise post hoc test was applied for comparing differences in each pair.

## 3. Results

Of 68 OI children, 20.6% (14 patients) had primary dentition, 53% (36 patients) had mixed dentition, and 26.4% (18 patients) had permanent dentition. Thus, primary teeth and permanent teeth were observed in 50 and 54 patients, respectively. There was no stainless-steel crown observed among participants.

### 3.1. Dentinogenesis Imperfecta

Dentinogenesis imperfecta was found in 32 patients (47.1%). Two patients with OI type I showed no features of DI. In the OI type III group, 18 patients presented with DI (51.4%), whereas this number in the OI type IV group was 14 (45.2%). No significant difference in DI prevalence was found between OI type III and type IV groups (*p* = 0.61). Twenty-six (52%) and twenty-four (44.4%) patients were diagnosed with DI in deciduous teeth and permanent teeth, respectively. The difference was not significant (*p* = 0.28) (Table 2).

**Figure 1:**
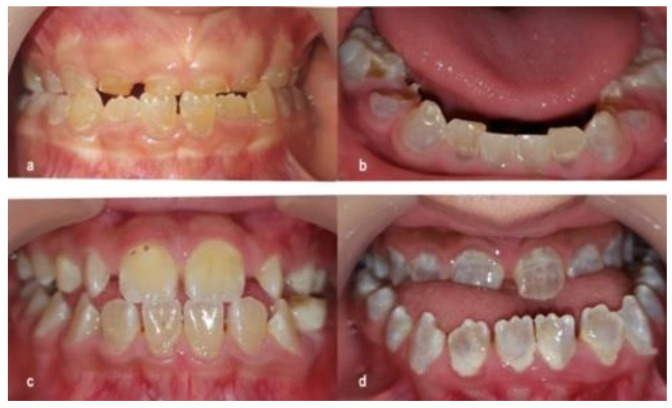
Tooth discoloration in DI patients: (**a**) yellow-brown discoloration in primary teeth; (**b**) opalescent-gray discoloration in primary teeth; (**c**) yellow-brown discoloration in permanent teeth; (**d**) opalescent-gray discoloration in permanent teeth.

The tooth discoloration varied among DI patients; however, it could be generally classified into two groups: yellow-brown and opalescent-gray. No significant difference in tooth discoloration between dentitions was found (*p* = 0.55). Cases of severe attrition were observed in primary teeth of the yellow-brown type only (Figure 1).

**Figure 2:**
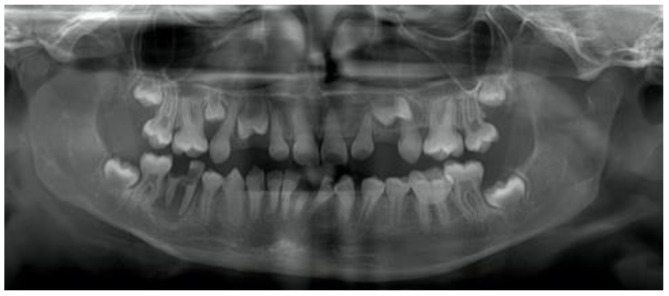
Pulp obliteration, low mineralization density, short roots, bulbous crowns, cervical constriction in panoramic radiography.

Radiography showed various degrees of pulpal obliteration among DI patients. Teeth with wide pulp or obliterated pulp were observed in 20 patients (62.5%). Other features such as poor mineralization density, short roots, bulbous crowns, and cervical constriction were also found (Figure 2).

### 3.2. Caries

The prevalence of caries in OI patients was 69.1%. In terms of OI types, two OI type I patients (100%) had caries. In the OI type III group, 25 patients (71.4%) presented with caries, whereas 20 patients (64.5%) in the OI type IV group were diagnosed with caries. No significant difference was found (*p* = 0.55). The prevalence of caries in primary teeth was 86%, significantly higher than that in permanent teeth (51.9%) (*p* < 0.001) (Table 2). 

Table 3 shows DMFT and dmft indices distributed by dentitions. The means of dft + DFT scores were highest at primary dentition as 7.5 ± 6.8, followed by mixed dentition 5.9 ± 6.0, and primary dentition 2.7 ± 2.6. A test of homogeneity of variances was also performed (*p* > 0.05). The ANOVA test showed a significant difference between three dentitions (*p* = 0.04). The dft+DFT score for primary dentition was significantly higher than that of permanent dentition via the LSD post hoc test (*p* = 0.045)

### 3.3. Occlusion, Eruption, Impacted and Missing Teeth

Among 68 children recruited, there were 14 patients with primary dentition and four patients with an uncompleted first molar relationship. A total of 50 patients were involved in the occlusion assessment. There was no class II malocclusion observed in three types of OI. Angle Class III malocclusion presented in most patients: 100% of type I patients, 89.2% in type III and 75% in type IV. In general, occlusion problems occurred more frequently in OI type III than in type IV patients in all findings. A high incidence of open bite and crossbite was expected, more frequent in anterior than posterior. No significant differences between OI type III and IV patients in all findings were observed, except posterior crossbite (Table 4). The prevalence of posterior crossbite in OI type III (71.4%) was significantly higher than that in OI type IV (25%) (*p* = 0.02).

Most patients (85.3%) achieved eruption within the normal range. However, five patients were noted with tooth 17 impacted, whereas six reported impactions of their 27 teeth. There was one case with missing teeth (upper second premolars) observed.

## 4. Discussion

This study was conducted on child subjects with severe OI types (type III and IV) to evaluate oral manifestations and caries status of OI children. The current study would provide valuable data for better dental management in OI patients.

### 4.1. Dentinogenesis Imperfecta

The prevalence of DI in the current study (47.1%) was higher than several studies, 19% in Norway [8], 20.4% in Egypt [9], 24.7% in Denmark [10], 42% in Sweden [18], but lower than other results in Italy (62.5%) [38] or in a previous Vietnamese study (61%) [39]. However, the indirect comparison across studies should be interpreted with caution since the OI type and age of participants were different between these studies. It was well reported that DI was more frequent in OI type III and IV than type I [40,41]. Therefore, studies in which participants were dominated by OI type I patients could give a lower estimate of the prevalence. For example, the Norway [8] and Denmark studies [10] involved 79% and 75% participants as OI type I, while the Vietnamese study [39] included 33.8% OI type I participants. If considering only OI type III and IV, the DI prevalences of these studies were 50% (Norway) [8], 25.6% (Egypt) [9], and 58.6% (Sweden) [18]. Moreover, in studies that included only a few cases in one OI type, the relative percentage might not be representative and reliable.

Consistent with other studies [9,13,18], the present investigation found a higher prevalence of DI in OI type III (51.4%) than OI type IV (45.2%), suggesting that DI is associated with the severity of OI. Since DI type I is derived from qualitative defects of collagen type 1 (COL1A1 and COL1A2) [1], the defect level of collagen type 1 might manifest relatedly in the severity of bone deformity and the dentin defect. The current study also revealed a higher prevalence of DI in the primary dentition (50%) than permanent dentition (44.4%). Our study observed some cases in which DI presented in the primary dentition; however, the permanent dentition was not affected. There have been no signs to predict in which patients this incident occurs [13]; early routine dental examination from primary dentition is necessary.

Tooth discoloration was found in both dentitions among DI children. O’Connell reported that yellow-brown type discoloration was more vulnerable to attrition than opalescent-gray type in primary dentition [13]. In this study, we also observed severe attrition cases of primary teeth in the yellow-brown type. It should be noted that most children with opalescent-gray type teeth had an open bite posteriorly or anteriorly, which assumes ineffective mastication. Therefore, the masticatory force exerted on teeth was not significant and attrition in these patients was not observed. It is unconfident to conclude that the attrition in the yellow-brown type was attributed solely to dentin itself or masticatory force. However, this finding provides a predictive value for early intervention in yellow-brown type patients to avoid vertical height loss. Stainless steel crowns are recommended to prevent the early attrition of primary molars [42]. Glass ionomer cement should be used for restorative treatment in non-stress teeth [43].

### 4.2. Caries

Another research attention was to investigate the caries status of children with OI. Our population’s caries prevalence was 69.1%, which was as high as the general non-OI population. Previous studies conducted on Vietnamese children reported caries prevalences from 75% to 90%. DT/dt indices were about 6 in primary teeth and 2.5 in permanent teeth, similar to common population studies [44,45,46]. It did not seem that OI increases the prevalence of caries. However, there was no control group in the current study; it is uncertain whether OI has a high risk of caries.

Typically, children with severe OI types (type III and IV) require routine medical care for bone fragility problems in hospitals, which assumes that dental checks were included. However, this study showed low scores of filled teeth (ft/FT = 0.2–1.3), indicating neglect of dental examination in OI children. Parents and general doctors should pay more attention to dental care to prevent caries and premature tooth loss.

### 4.3. Occlusion, Eruption, Impaction and Missing Teeth

The incidence of Class III malocclusion in our OI group was high (89.2% in type III and 75% in type IV), compared to 15.5% reported in a normal Vietnamese population [47]. Similar findings were reported in different populations [13,18]. Many factors might play a part in the etiology of malocclusion, such as inhibition in maxillary growth [21], mandibular protrusion [19], maxillary hypoplasia or mandibular hyperplasia [48]. Crossbite and open bite, found commonly in this study, were not unexpected. An underdeveloped nasomaxillary complex could translate to dental occlusion, causing Angle Class III malocclusion, crossbite and open bite [49,50]. Posterior crossbite could be derived from the abnormal development of alveolar bone, which can enlarge the tongue’s space [13]. The high incidence of Class III malocclusion, crossbite, and open bite suggests the need for orthodontic treatments and orthognathic surgeries in these OI patients, reflecting on different surgical approaches [51,52]. A few cases reported promising orthognathic surgery results in correcting skeleton malocclusion in mild OI patients [48,53]. However, in OI cases, orthognathic surgery is still questionable due to concerns about potential bleeding and bisphosphonate side effects [50,53]. Orthodontic complications include excessive mechanical force [42], subacute bacterial endocarditis, or invasion when debonding [53]. Treatments for class III correction by orthodontic and orthopedic jaw movement are most effective at the age of 7 [54], so the OI children should see orthodontists before that age.

About eruption, most of all OI patients had an average eruption. Impacted teeth and missing teeth were rarely found in OI type III patients.

### 4.4. Limitations

Our study evaluated oral perspective on severe OI types (type III and IV), only with no control group. Periodontal and orthodontic indices were not mentioned in this current investigation. A further study with a control group in the design including OI type I patients and more dental indices is needed to provide insight and an extensive picture of oral manifestations and oral health care status of OI children in Vietnam.

## 5. Conclusions

DI was the most prominent oral feature in OI patients; yellow-brown type discoloration was more vulnerable to attrition than opalescent-gray type in the primary dentition. OI did not increase the prevalence of caries. The high incidence of Class III malocclusion, open bite, and crossbite suggests a need for orthodontic treatments and orthognathic surgeries in these OI patients. Early and regular dental examination is needed for preventing caries and premature tooth loss.

## Figures and Tables

**Table 1:** Gender and age distribution in each OI type.

OI Type	Total	Gender		Age (year)		
		Male	Female	3–5	6–12	13–17
Type I	2	2	0	0	0	2
Type III	35	24	11	2	21	12
Type IV	31	15	16	8	19	4

**Table 2:** Prevalence of dentinogenesis imperfecta and caries in OI patients.

		OI Type				Dentition		
	All	I (n = 2)	III (n = 35)	IV (n = 31)	*p* ^a^	Primary (n = 50)	Permanent (n = 54)	*p*-value
DI	47.1%	0%	51.4%	45.2%	0.61	52%	44.4%	0.44
Yellow-brown	-	-	-	-	-	13	14	0.55
Opalescent-gray	-	-	-	-	-	13	10	
Caries	69.1%	100%	71.4%	64.5%	0.55	86%	51.9%	<0.001

^a^ Chi-square test, for comparison between group III and IV only.

**Table 3:** DMFT and dmft scores distributed by dentitions.

Dentition	dt	ft	dft	DT	FT	DFT	Dft + DFT	*p*-Value
Primary (n = 14)	6.2 ± 5.9	1.3 ± 2.8	7.5 ± 6.8	-	-	-	7.5 ± 6.8	0.04
Mixed (n = 36)	4.4 ± 5.5	0.2 ± 1.1	4.6 ± 5.7	1.1 ± 1.9	0.2 ± 0.7	1.3 ± 2.1	5.9 ± 6.0	
Permanent (n = 18)	-	-	-	2.5 ± 2.5	0.2 ± 0.5	2.7 ± 2.6	2.7 ± 2.6	

dt (DT): decayed teeth, ft (FT): filled teeth.

**Table 4:** Occlusion problems, impacted and missing teeth in OI patients.

	Findings	Type I	Type III	Type IV	*p*^a^-Value
		n = 2	n = 28	n = 20	
Malocclusion	Class I	0	10.8%	25%	0.19
	Class II	0	0	0	
	Class III	100%	89.2%	75%	
Open bite	Anterior	100%	42.9%	40%	0.84
	Posterior	100%	39.3%	15%	0.07
Cross bite	Anterior	100%	82.1%	60%	0.09
	Posterior	100%	71.4%	25%	0.002
		n = 2	n = 35	n = 31	
Eruption	Early	0	5.7%	3.2%	0.63
	Normal	2	74.3%	96.8%	0.01
	Late	0	20%	0	-
Impacted tooth n, (%)	Tooth 17	0	5, (14.3%)	0	
	Tooth 27	2	4, (11.4%)	2, (6.5%)	
Missing tooth		0	1, (2.9%)	0	

^a^ for comparison between group III and IV.

## Data Availability

The data that support the findings of this study are available from the corresponding authors, upon reasonable request.
